# Association of household food insecurity with sociodemographic factors and obesity in US youth: findings from the National Health and Nutrition Examination Survey 2017–2018

**DOI:** 10.3389/fpubh.2024.1387638

**Published:** 2024-07-17

**Authors:** Amin Mokari-Yamchi, Amir Hossein Faghfouri, Samira Gholami, Elyas Nattagh-Eshtivani, Shahsanam Gheibi

**Affiliations:** ^1^Maternal and Childhood Obesity Research Center, Urmia University of Medical Sciences, Urmia, Iran; ^2^Department of Nutrition, Food Sciences and Clinical Biochemistry, School of Medicine, Social Determinants of Health Research Center, Gonabad University of Medical Science, Gonabad, Iran

**Keywords:** food insecurity, obesity, youth, NHANES, sociodemograhic factors

## Abstract

**Background:**

The objective is to determine the prevalence of household food insecurity (HFI) based on sociodemographic factors and their relationship to obesity in youth.

**Methods:**

The study included a sample of 1,962 youth (aged 6–18) from the National Health and Nutrition Examination Survey (NHANES). The US Household Food Security Survey Module is used to measure food security over the past 12 months. Logistic regression models were used to estimate adjusted odds ratios (ORs) while controlling for covariates.

**Results:**

In total, 27.4% of the individuals surveyed experienced HFI. Youth from food insecure households were more likely to be obese (adjusted odds ratio [aOR]: 1.59 [95% confidence interval: 1.19–2.13]) and also having abdominal obesity (aOR: 1.56 [95% CI: 1.19–2.03]). however, factors such as non-Hispanic ethnicity, having a Head of household with a college degree, and households with an income exceeding 350% of the poverty line were associated with a reduced risk of facing HFI.

**Conclusion:**

Hispanic individuals, households with lower parental education levels, and lower family incomes, are disproportionately affected by food insecurity. Furthermore, HFI has been associated with an increased risk of overweight and abdominal obesity among youth. Addressing FI requires targeted policies and interventions that prioritize vulnerable groups.

## Introduction

Household Food insecurity (HFI) is defined as the inconsistent economic and physical access to obtain enough safe and nutritious food to lead an active and healthy lifestyle (1). It encompasses a broad spectrum, ranging from concerns about an inadequate food supply to more sever levels of hunger (2). According to a recent report by the Food and Agriculture Organization (FAO), approximately 735 million people worldwide are still struggling with hunger on a daily basis, indicating the widespread and persistent nature of the issue (1). Additionally, the 2022 report from the US Department of Agriculture (USDA) estimates that roughly 17 million household in the US are affected by food insecurity (3).

Numerous studies have demonstrated the potential consequences of HFI, particularly on children and adolescents, which may include inadequate nutrient intake and various physical and mental health issues, including anemia, asthma, cognitive impairments, as well as behavioral concerns such as aggression and anxiety (4–6). HFI is a strong predictor of childhood malnutrition due to its close connection with the accessibility and quality of food in a household. Various studies have also found that food insecurity can contribute to both underweight and overweight among young people, making it a potential barrier to preventing and treating obesity (7–9).

Recent findings from National Health and Nutrition Examination Survey (NHANES) reveal a concerning trend of increasing obesity rates among youth from 1999 to 2000 to 2015–2016. Conducting research to identify risk factors contributing to adolescent obesity is essential for identifying potential areas of intervention, such as lifestyle modifications and socioeconomic interventions. Recent studies, like the one conducted by Jun et al. (10), have found an correlation between food insecurity and older age, as well as lower household income and educational background.

However, there is conflicting evidence on the relationship between food insecurity and markers of metabolic syndrome. For example, Holben and Taylor analyzed NHANES data and found that youth from food secure families had higher high-density lipoprotein (HDL) values (11). In contrast, Fulay et al. conducted a study among youth aged 12–17 and did not find any significant associations between HFI and Body Mass Index (BMI) for age Z-score, total cholesterol, HDL-C, fasting triglycerides, LDL-C, or fasting plasma glucose (12).

Also an earlier study conducted by Fleming et al. showed no significant associations between food insecurity and obesity among US youth. Nevertheless, it is important to note that the study used a BMI threshold of ≥95th percentile to define obesity, and a BMI percentile range of >5th percentile to <95th percentile for the non-obese group (13). Despite previous research, there is still conflicting evidence regarding the connections between food insecurity and obesity among youths. Thus, our aim in this study, using NHANES data, is to examine the association between food security status, obesity, metabolic syndrome biomarkers, and sociodemographic factors among youths.

## Materials and methods

We conducted our study using data from the 2017–2018 NHANES, a robust cross-sectional survey conducted by Centers for Disease Control and Prevention (CDC). NHANES employs a meticulous multistage probability sampling method to select participants, ensuring that the non-institutionalized U.S. civilian population is adequately represented. The survey results are carefully weighted to provide an accurate reflection of population demographics. More details on the sampling method can be found on the NHANES website.^1^ In this study, all youth who were 6–18 years of age were eligible for inclusion. The total sample size we analyzed consisted of 1,962 participants.

### Sociodemographic variables

In our analysis, we considered various demographic characteristics, including age, gender, ethnicity, and three indicators of socioeconomic status: family income to poverty guidelines ratio (FIPR), highest level of education received by the head of the family, and marital status. FIPR was divided into three categories: low income (0–1.3), middle income (1.3–3.5), and high income (>3.5–5). To determine obesity status, we utilized age- and sex-specific BMI percentiles calculated as weight in kilograms divided by height in meters squared, based on the 2000 CDC growth charts. A BMI ≥85th and < 95th percentile was considered overweight, while a BMI ≥95th percentile was classified as obese. Additionally, we assessed abdominal obesity by using a waist-to-height ratio (WHtR) threshold of ≥0.5.

### Food security measurement

The assessment of HFS in the NHANES study involved the use of a validated 18-item questionnaire developed by the USDA (14). This questionnaire evaluates the food security status of the household over the course of the past 12 months. Based on the responses from the Household Food Security Scale (HFSS), households were categorized into two groups: (1) food secure (fully and marginal food secure) and (2) food insecure (low and very low food secure).

### Laboratory tests

Laboratory tests were performed directly in CDC laboratories in accordance with established protocols using blood samples collected by trained phlebotomists at the Mobile Examination Center (MEC). Enzymatic methods were used to measure serum total cholesterol, LDL-C, and HDL-C levels. Fasting glucose levels were assessed through hexokinase enzymatic and immunoenzymatic assay methods. More details on the measurement of laboratory tests can be found on the NHANES website (see text footnote 1).

### Statistical analysis

The statistical analysis was conducted using SPSS software (V 22; SPSS Inc., Chicago, IL), and *p* < 0.05 were considered statistically significant. An independent sample t-test was performed to determine the statistical differences in serum health variables, between youth from food secure and food insecure households. Logistic regression analysis was employed to examine the relationship between sociodemographic factors, obesity status, and HFI and odds ratio (OR) with 95% confidence intervals (CIs) were reported. Adjustment for potential confounding variables, such as age, sex, race, household income, and the education and marital status of the head-of-household, was performed during the analysis.

## Results

The study included 1,962 participants between the ages of 6 and 18. 27.4% of participants were HFIs. The sample was ethnically diverse; 32.9% were Hispanic, 37.9% were non-Hispanic white individuals, and 29.2% were non-Hispanic black individuals. Additionally, 50.2% of the participants were male (Table 1).

**Table 1:** Sociodemographic characteristics of the youth by food security status, 2017–2018 NHANES.

Variable	Sample size	Food secure	Food insecure
Overall	1,962	1,424 (72.6)	538 (27.4)
Gender			
Male Female	984 978	725 (50.9) 699 (49.1)	259 (48.1) 279 (51.9)
Age group, y			
6–9 10–13 14–18	654 622 686	449 (31.5) 467 (32.8) 508 (35.7)	205 (38.1) 155 (28.8) 178 (33.1)
Race/ethnicity			
Hispanic Non- Hispanic white Non- Hispanic Black	511 589 453	312 (21.9) 469 (32.9) 312 (21.9)	199 (43.3) 120 (26.1) 141 (30.7)
Obesity status			
Normal Over weight Obese	1,126 335 501	873 (61.3) 228 (16) 323 (22.7)	263 (47) 107 (19.9) 178 (33.1)
Abdominal obesity status			
WHtR <0.5 WHtR ≥0.5	1,180 710	903 (66.1) 464 (33.9)	277 (53) 246 (47)
Head-of-household gender			
Male Female	871 1,091	674 (47.3) 750 (52.7)	197 (36.6) 341 (63.4)
Head-of-household education level			
< high school High school College graduate ≤	376 1,083 431	220 (16.2) 747 (54.8) 395 (29)	156 (29.5) 336 (63.6) 36 (6.8)
Head-of-household marital status			
Married Single	1,336 573	1,028 (74.2) 357 (25.8)	308 (58.8) 216 (41.2)
Income (%FIPR)			
<130% 130–349% ≥350%	695 714 395	387 (29.7) 546 (41.8) 372 (28.5)	308 (61.7) 168 (33.7) 23 (4.6)

Values are frequency (%). FIPR, family income to poverty level ratio; NHANES, National Health and Nutrition Examination Survey; WHtR, waist-to-height ratio,

Table 2 displays the findings from logistic regression analyses investigating the relationship between variables related to HFI. Non-Hispanic Black and White households were significantly less likely to be food insecure than Hispanic households (aOR: 0.5 [95% confidence interval (CI): 0.36–0.69], and 0.62 [95% CI: 0.45–0.86], respectively). The likelihood of food insecurity was higher among youth who lived with a single parent (aOR, 1.44 [95% CI, 1.06–1.95]). Additionally, having a head of household with a college degree compared to less than a high school education was associated with a lower odds ratio of HFI (aOR, 0.34 [95% CI, 0.2–0.6]). In addition, there was a decrease in the likelihood of food insecurity among youth living in households that had an income equal to or greater than 350% of the poverty line (aOR: 0.25 [95% CI: 0.14–0.47]). Additionally, the probability of food insecurity decreased for youth in households with an income at or above 350% of the poverty line (aOR: 0.25 [95% CI: 0.14–0.47]), while those below 130% of the poverty line had a higher risk of food insecurity (aOR: 2.43 [95% CI: 1.8–3.2]).

**Table 2:** Logistic regression of household food insecurity depending on sociodemographic factors, 2017–2018 NHANES.

	Food insecure	
Variable	Unadjusted^1^	Adjusted^2^
Gender		
Female Male	1 0.89 (0.73–1.09)	1 0.88 (0.68–1.13)
Age group, y		
6–9 10–13 14–17	1 0.72 (0.57–0.93) 0.76 (0.6–0.97)	1 0.78 (0.58–1.07) 0.78 (0.57–1.06)
Race/ethnicity		
Hispanic Non-Hispanic white Non-Hispanic Black	1 0.4 (0.3–0.52) 0.71 (0.54–0.92)	1 0.5 (0.36–0.69) 0.62 (0.45–0.86)
Head-of-household gender		
Male Female	1 1.55 (1.27–1.9)	1 0.84 (0.62–1.12)
Head-of-household education level		
<High school High school College graduate ≤	1 0.63 (0.49–0.8) 0.13 (0.08–0.19)	1 0.9 (0.66–1.23) 0.34 (0.2–0.6)
Head-of-household marital status		
Married Single	1 2 (1.63–2.49)	1 1.44 (1.06–1.95)
Income (%FIPR)		
130–349% <130% ≥350%	1 2.58 (2.05–3.25) 0.2 (0.12–0.31)	1 2.43 (1.8–3.2) 0.25 (0.14–0.47)

FIPR, family income to poverty level ratio; NHANES, National Health and Nutrition Examination Survey. Model^1^: crude mode. Model^2^: adjusted for all variables shown in the table. Different from the food insecure category.

As shown in Table 3, there were no significant differences in average levels of total cholesterol, blood glucose, and LDL cholesterol between youth from food secure and food insecure households. However, participants from food secure households had higher average levels of HDL cholesterol and lower levels of hsCRP compared to those from food insecure households (*p* < 0.01).

**Table 3:** The health measures of the youth by household food security status, NHANES 2017–2018.

Variable	Food secure	Food insecure	*P*-value
Total cholesterol, mg/dL	157.36 (28.26)	155.96 (26.98)	0.35
HDL cholesterol, mg/dL	54.43 (11.8)	52.62 (11.32)	0.005
LDL cholesterol, mg/dL	88.36 (24.47)	87.95 (25)	0.89
Fasting glucose, mg/dL	97.69 (7.83)	99.75 (13.75)	0.24
HS-CRP (mg/L)	1.59 (3.85)	2.11 (5.26)	0.032
Systolic blood pressure (mmHg)	105.39 (10.11)	105.43 (9.96)	0.94
Diastolic blood pressure (mmHg)	55.6 (18.55)	54.66 (18.83)	0.38

Values are Mean (SD). HDL-C, high-density lipoprotein cholesterol; LDL-C, low-density lipoprotein cholesterol; HS-CRP, high-sensitivity C-reactive protein; NHANES, National Health and Nutrition Examination Survey.

**Figure 1:**
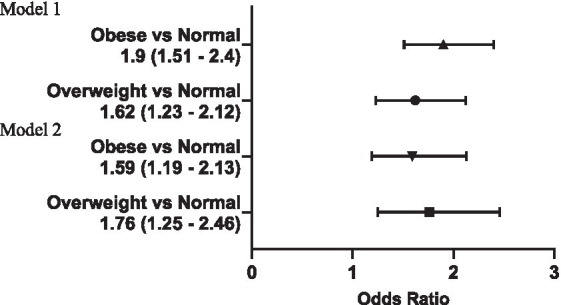
Association of household food insecurity with risk of overweight and obesity in youth 6 to 18 y; OR with 95% CI. Model 1 = Unadjusted Model; Model 2 = adjusted for age, sex, race, household income and Head-of-household marital and education status.

Figure 1 displays the association between HFI and the risk of overweight and obesity in youth, both in crude and adjusted models. The logistic regression analysis, after accounting for confounding variables, indicated that youth from food insecure households had 1.59 and 1.76 times greater odds of being overweight and obese, respectively.

**Figure 2:**
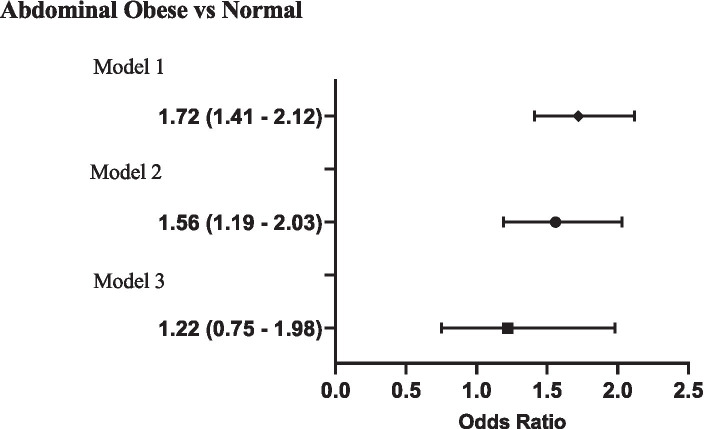
Association of household food insecurity with risk of abdominal obesity (WHtR≥0.5) in youth aged 6 to 18 y; OR with 95% CI. Model 1 = Unadjusted Model; Model 2 = adjusted for age, sex, race, household income and Head-of-household marital and education status; Model 3= adjusted for age, sex, race, household income and Head-of-household marital and education status and BMI.

Furthermore, Figure 2 showed that food insecurity was linked to higher odds of abdominal obesity in the unadjusted model (OR: 1.72 [95% CI: 1.41–2.12]), a relationship that persisted even after adjusting for various factors (aOR: 1.56 [95% CI: 1.19–2.03]). However, when BMI was included in the adjusted model alongside other confounders, the association was no longer statistically significant (aOR: 1.22 [95% CI: 0.75–1.98]).

## Discussion

This study investigated the relationship between HFI and anthropometric measurements, metabolic syndrome indicators, and sociodemographic factors among youth in the United States. The data revealed that HFI was associated with an increased risk of overweight, obesity, and central obesity in youth. Consistent with these findings, previous studies have also shown a clear connection between HFI and obesity (13, 15). For example, Ortiz-Marrón et al. found that children experiencing HFI had nearly double the prevalence of childhood overweight and obesity compared to those with access to HFS (16), However, some other studies have not found such a connection (12, 17). The coexistence of obesity and FI has raised concerns among researchers due to the apparent contradiction that individuals with limited access to food can still become obese. There are several potential factors that may contribute to the association between food insecurity and youth obesity. These factors include the consumption of energy-dense foods in excess (18), overeating during periods of food abundance (19), and reduced family support and attention toward making healthy nutritional choices (20, 21).

Youth from food secure households had significantly higher levels of serum HDL-C compared to those from food insecure households. However, there were no significant differences in other metabolic syndrome indicators like blood pressure, total and LDL-C, and fasting glucose levels between the two groups. These findings align with Holben et al.’s study, which showed that adolescents facing marginal food security or food insecurity were more likely to have increased central adiposity, be overweight or obese, and have lower HDL-C levels compared to those with full food security (11). The higher levels of HDL-C observed in youth from food secure households may be attributed to various factors, including greater engagement in physical activity, fewer limitations in resources, or residing in neighborhoods that offer better infrastructures for physical activity (22).

Youth who come from food insecure households have been found to have significantly higher levels of hs-CRP in their serum. This trend has also been observed in previous studies with food insecure adults (23). Elevated hs-CRP levels during childhood and adolescence can predict future cardiovascular disease risk (24). Food insecurity may lead to increased inflammation through various mechanisms, including poor diet quality due to inadequate access to nutritious foods and the stress of food scarcity or uncertainty about meal availability (23, 25).

The correlations identified between HFI and sociodemographic factors are consistent with the findings reported in previous studies (13, 26–28). Households with parents who have lower education levels, lower family incomes, and single parental status showed a higher prevalence of food insecurity. An increase in parents’ education level can lead to more job opportunities and higher income which provides the power to buy food (26). Additionally, higher parental education levels have been linked to improved awareness, attitudes, and actions regarding family nutrition (29, 30).

Our findings also indicated that non-Hispanic youth are less likely to be food insecure compared to Hispanic youth. This supports the findings of Fleming et al., who reported a high prevalence of food insecurity among Hispanic youth (13). Hispanic youth face various challenges related to acculturation, including adapting to new norms and family dynamics, as well as economic factors such as low-income status and neighborhood isolation. These factors collectively contribute to their increased risk of food insecurity. Additionally, immigration status and associated difficulties may further exacerbate food insecurity among Hispanic youth and their families. Concerns about deportation can act as barriers to accessing government assistance programs, making them more susceptible to food insecurity (31–33).

Implementation of targeted, specific policies like the Supplemental Nutrition Assistance Program (SNAP) in the US, which offers food assistance to low-income individuals and families, as well as initiatives like the National School Lunch Program and the Special Supplemental Nutrition Program for Women, Infants, and Children (WIC), which provide nutritious meals and support to children and pregnant or postpartum women from low-income households, can play a crucial role in addressing these disparities. By focusing on improving access to healthy and nutritious foods for youth from food-insecure households, targeted nutrition programs can help reduce the heightened risk of overweight, obesity, and central obesity associated with HFI.

The main limitation of the current study is its cross-sectional design, which does not provide conclusive evidence of causality. To enhance our comprehension of the connection between sociodemographic factors, food security and health related problems in youth, it is imperative to conduct longitudinal studies.

## Conclusion

Food insecurity disproportionately affects specific population groups, including Hispanic individuals, households with lower parental education levels, lower family incomes, and single parental status. Furthermore, youth living in households with food insecurity have a higher likelihood of being overweight and having abdominal obesity. It is crucial to address food insecurity by implementing policies and interventions that focus on improving economic stability, parental education, and household income. The study highlights the importance of targeted policies and programs, in addressing food insecurity and its associated health risks among vulnerable youth populations. These measures have the potential to alleviate the negative health consequences associated with food insecurity, particularly among vulnerable populations.

## Data availability statement

The original contributions presented in the study are included in the article/supplementary material, further inquiries can be directed to the corresponding authors.

## Ethics statement

The NHANES protocol has been reviewed and approved by the National Center for Health Statistics research ethics review board. The studies were conducted in accordance with the local legislation and institutional requirements. Written informed consent for participation in this study was provided by the participants’ legal guardians/next of kin.

## Author contributions

AM-Y: Conceptualization, Formal analysis, Writing – original draft, Writing – review & editing. AF: Data curation, Methodology, Writing – original draft. SaG: Writing – original draft. EN-E: Writing – review & editing. ShG: Writing – review & editing.

## Footnotes

^1^ https://www.cdc.gov/nchs/nhanes/index.htm
